# Book Reviews

**Published:** 1917-06

**Authors:** 


### BOOK REVIEWS

### IMPOTENCY, STERILITY AND ARTIFICIAL IMPREGNATION.

Frank P. Davis, M.D., St. Louis, C. V. Mosby Co.

This small book is well written and presents in a concise, clear fashion what you are led to believe the author knows. It contains thirteen chapters, dealing with "The Sexual Instinct", "The Sense of Smell", "The Voice and Sense of Hearing", "The Sense of Sight", "Impotency", "Psychic Impotency", "Masturbation and Emissions", "Treatment of Impotency", "Race Suicide", "Sterility", "Treatment of Sterility", "Artificial Impregnation", "Therapeutics". This book is evidently written for guide only and it fits its place admirably.

---

### MEDICINE AND SURGERY.

By Philip Shrainka, M.D., Editor-in-chief. Published Monthly, 30 cents a copy, $3.00 a year. Medicine and Surgery Publishing Company, St. Louis, Mo.

We welcome No. I, Vol. I. of Medicine and Surgery as a worthy representation, judging by the first issue. In the announcement it says that the policy will be to have throughout the year a large number of special articles on Surgery, Genito-Urinary Diseases, Gynecology and Obstetrics. The contributors to the first number are Wm. Pearce Cones, M.D.; Kellog Speed, M.D.; John M. Swan, M.D.; J. Shelton Horsley, M.D.; H. A. McKnight, M.D.; Vilray P. Blair, M.D. F. A. C. S., James K. Young, M.D. F. A. C. S. and many more.

